# Development and patterns of acute-on-chronic liver failure in patients with cirrhosis and acute kidney injury

**DOI:** 10.1016/j.jhepr.2026.101734

**Published:** 2026-01-12

**Authors:** Susan Fischer, Martin Sebastian McCoy, Marta Fiocco, Annarein Kerbert, Eduardo Cervantes-Alvarez, Jan Hähner, Michael Praktiknjo, Maximilian Joseph Brol, Frank Erhard Uschner, Lena Wolters, Stefan Zeuzem, Josune Cabello, Kai-Henrik Peiffer, Jeetindra Balak, Sesmu Arbous, Jeroen Nieuwenhuizen, David van Westerloo, Anton Jan van Zonneveld, Jonel Trebicka, Minneke Coenraad

**Affiliations:** 1 Department of Gastroenterology and Hepatology, Leiden University Medical Center, Leiden, The Netherlands; 2 Department of Internal Medicine B, University of Münster, Münster, Germany; 3 Department of Internal Medicine I, Goethe University, Frankfurt am Main, Germany; 4 Department of Biomedical Data Science, Leiden University Medical Center, Leiden, The Netherlands; 5 Mathematical Institute Leiden University, Leiden, The Netherlands; 6 Princess Maxima Center for Pediatric Oncology, Utrecht, The Netherlands; 7 Department of Internal Medicine, Leiden University Medical Center, Leiden, The Netherlands; 8 Department of Intensive Care, Leiden University Medical Center, Leiden, The Netherlands; 9 The Einthoven Laboratory for Vascular and Regenerative Medicine, Leiden University Medical Center, Leiden, The Netherlands; 10 Department of Anesthesiology, Intensive Care and Pain Medicine, University Hospital Münster, Münster, Germany

**Keywords:** AKI, acute kidney injury, ACLF, acute-on-chronic liver failure, HRS-AKI, Hepatorenal Syndrome Acute Kidney Injury, albumin, respiratory failure, circulatory failure, cirrhosis

## Abstract

**Background & Aims:**

The prevalence and evolution of acute-on-chronic liver failure (ACLF), particularly extrarenal organ failures, in patients with cirrhosis and acute kidney injury (AKI) are not well characterized. This study investigated the development and progression of ACLF in patients with cirrhosis who develop AKI, aiming to improve understanding of disease course during the critical period following AKI onset.

**Methods:**

We conducted a retrospective cohort study of hospitalized patients with cirrhosis and AKI at two tertiary centers between 2010 and 2023. Data on AKI etiology, treatment, ACLF development and progression, and survival were collected. Multivariable regression models were used to assess associations between baseline and AKI-related characteristics, ACLF outcomes, and mortality.

**Results:**

A total of 672 patients (71% male) were included. AKI progression or non-response to therapy occurred in 47% of patients. ACLF was present at the time of AKI diagnosis in 406 patients (60%); among these, 106 (26%) experienced ACLF progression, predominantly involving renal, respiratory, and circulatory failure. Of the 266 patients without ACLF at AKI diagnosis (40%), 101 (38%) subsequently developed ACLF, most commonly with renal, respiratory, and liver failure. In multivariable analysis, patients with hepatorenal syndrome-AKI (HRS-AKI) or other/mixed AKI etiologies had a higher risk of ACLF development compared to those with pre-renal AKI (odds ratio [OR] 9.67, 95% CI 3.96–23.57; OR 4.98, 95% CI 1.78–12.95, respectively). HRS-AKI and AKI stage 2 were independently associated with ACLF progression after adjustment for MELD score and relevant clinical risk factors (OR 2.31, 95% CI 1.08–4.95; OR 2.35, 95% CI 1.03–5.36). The cumulative incidence of death was 47% at 90 days after AKI diagnosis (95% CI 44–51).

**Conclusions:**

Patients with cirrhosis who develop AKI are at high risk of ACLF development and mortality. Respiratory failure is the most frequent extrarenal organ failure among patients who develop ACLF or experience ACLF progression.

**Impact and implications:**

This study highlights the significant risk of acute-on-chronic liver failure development in patients with cirrhosis and acute kidney injury (AKI), particularly in those with hepatorenal syndrome-AKI or other/mixed types of AKI. Given the high short-term mortality observed, early recognition and risk stratification of AKI in cirrhosis are crucial. These findings are particularly relevant for hepatologists, nephrologists, and intensivists, as they underscore the need for improved therapeutic strategies targeting AKI non-responders. Future prospective studies should not only explore targeted interventions to improve outcomes in this high-risk population but also aim to elucidate the underlying pathophysiology driving AKI progression and acute-on-chronic liver failure development in patients with cirrhosis and AKI.

## Highlights

• AKI in cirrhosis is associated with short-term ACLF development and mortality.
• HRS-AKI was associated with an increased risk of ACLF development and progression.
• Respiratory and circulatory failure are common organ failures in patients with AKI and ACLF.
• Early identification and new strategies are needed to improve outcomes in patients with cirrhosis and AKI.

## Graphical abstract

**Figure:**
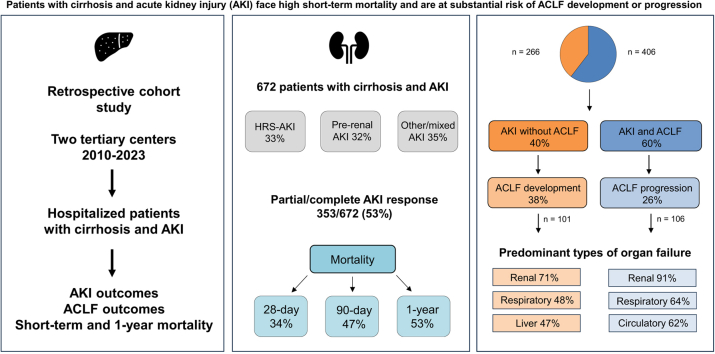

## Introduction

Acute kidney injury (AKI) is frequently encountered in patients with cirrhosis. AKI occurs in up to 50% of hospitalized patients with a decompensating event, and it is associated with a poor prognosis.[1], [2], [3] The spectrum of causes of AKI includes pre-renal AKI, hepatorenal syndrome-AKI (HRS-AKI), which is marked by severe AKI resulting from renal hypoperfusion and systemic inflammation in decompensated cirrhosis, intra-renal, and post-renal AKI.^4^^,^^5^ Treatment differs depending on the etiology of AKI.^1^^,^^6^^,^^7^

AKI and renal dysfunction can lead to renal failure, one of the six types of organ failure in acute-on-chronic liver failure (ACLF). ACLF is a syndrome characterized by the onset of organ failure in patients with acutely decompensated cirrhosis and is associated with high short-term mortality, depending on the extent of organ failures.[8], [9], [10], [11] ACLF often involves renal dysfunction or failure. Therefore, AKI emerges as a significant determinant of the short-term mortality risk associated with ACLF.^4^^,^^8^^,^^9^^,^^12^

Current treatment of AKI is multilayered and should take into account both etiology and disease stage.^13^ For example, cases of respiratory failure have been reported in patients treated with terlipressin among those with HRS-AKI and more advanced grades of ACLF.^14^ Renal replacement therapy (RRT) can be applied in patients with cirrhosis and HRS-AKI who do not respond to treatment as a bridge to liver transplantation.^1^ However, patients who require RRT and are not suitable candidates for liver transplantation have high mortality rates.^15^ Therefore, a balanced treatment approach is crucial in managing AKI in patients with cirrhosis.

The effectiveness of the European Association for the Study of the Liver (EASL) AKI management algorithm in clinical practice has recently been validated.^16^ Nevertheless, long-term survival remains poor, especially in patients with HRS-AKI who are not eligible for liver transplantation, even if they initially respond to treatment.^17^ While several studies have been conducted on AKI in cirrhosis, much of the existing knowledge focuses on select patient groups. Previous studies have shown that different stages of AKI influence renal outcomes and survival, and that early recognition of ACLF in patients with cirrhosis and AKI is crucial.^18^ However, in unselected patients with cirrhosis and AKI, the incidence of ACLF, and particularly the development of extrarenal organ failures is unknown. Moreover, the relationship between AKI etiology and stage, and the development of extrarenal organ failure has not previously been studied. Understanding the disease course during the critical period after AKI onset is essential to identify risk factors for ACLF development or progression and improve survival in patients with decompensated cirrhosis and AKI.

This study aims to investigate ACLF patterns in patients with cirrhosis diagnosed with AKI, with a specific focus on extrarenal organ failures. Secondly, we aim to identify potential risk factors and explore their relationship with AKI non-response/progression, ACLF development/progression and mortality.

## Patients and methods

A retrospective cohort study was performed in the Leiden University Medical Center (LUMC) and University Hospital Münster (UKM), both tertiary referral centers with liver transplant facilities. Data were collected from adult patients with cirrhosis who were admitted to the hospital for AKI or who developed AKI during the course of hospitalization. Only single AKI episodes per patient were included. Data were extracted from digital patient medical records between 2010-2023. Baseline characteristics at the time of hospitalization were obtained. The following data regarding disease course during hospitalization were collected: AKI characteristics, types of AKI therapy, AKI outcomes and duration of hospital admission. Types of organ failure, organ failure scores and grades of ACLF were also collected. Follow-up data were analyzed at 28 days, 90 days and 1 year.

Inclusion criteria were hospitalized patients aged ≥18 years with cirrhosis who met the diagnostic criteria for AKI according to the International Club of Ascites,^5^ including those admitted before 2015. The exclusion criteria consisted of the presence of chronic kidney disease, long-term dialysis and a history of liver or kidney transplantation. Patients with AKI in the presence of underlying chronic kidney disease were excluded to avoid misclassification of renal failure in ACLF.

Primary endpoints were the presence or development of ACLF, including types of organ failure, in patients with AKI within 1 year of follow-up. Secondary outcomes were resolution or progression of AKI during hospitalization, liver transplantation within 1 year of follow-up and 28-day, 90-day and 1-year mortality.

### Diagnostic and outcome definitions

The diagnosis of cirrhosis was based on a combination of clinical, laboratory, histological and imaging findings. For the diagnosis of AKI, the criteria outlined by the International Club of Ascites (ICA) (2015) were used, which included either an increase in serum creatinine (sCr) of ≥26.5 μmol/L within a 48-hour period or an increase in sCr of ≥50% from a known or presumed baseline value that has occurred within the preceding 7 days.^5^ Due to the retrospective nature of the study, it was not possible to consistently retrieve data on urine output. Therefore, the 2015 ICA criteria were adhered to, rather than the more recent Acute Disease Quality Initiative (ADQI) and ICA joint consensus meeting criteria, which incorporate urine output measurements in the diagnosis of AKI.^19^ Baseline sCr was defined as the most recent measurement obtained within 3 months prior to AKI onset. In patients without a prior measurement, the admission sCr was used as the baseline for analysis. Stage 1 AKI indicated an increase of ≥26.5 μmol/L or ≥1.5-2 times the baseline level, stage 2 denoted an increase of >2-3 times the baseline level. Stage 3 was characterized by a rise in serum creatinine >3 times the baseline level, or ≥353.6 μmol/L or the initiation of RRT.

Progression of AKI was determined by either advancement to a higher stage of severity of AKI and/or requirement for RRT. No therapeutic response was defined as no regression of AKI stage. Partial response was defined as regression of AKI severity with a reduction in sCr to ≥26.5 μmol/L above baseline. Complete response was defined as a return of sCr to <26.5 μmol/L above baseline.^5^

### Etiology of AKI

In line with established criteria,^5^ prerenal AKI was diagnosed in cases of documented fluid loss or bleeding, with subsequent renal improvement post intravenous fluid administration. According to revised ICA criteria, HRS-AKI was diagnosed in patients with ascites not responding to volume expansion with albumin and diuretic withdrawal for 2 days and absence of shock, without nephrotoxic drug use, absence of renal structural damage and no ultrasonographic abnormalities.^4^ Acute tubular necrosis (ATN)-AKI was diagnosed based on a clinical history consistent with ischemic or nephrotoxic AKI, alongside failure to respond to volume administration.^16^^,^^20^^,^^21^ Post-renal causes of AKI are caused by obstructive factors that contribute to renal dysfunction and were diagnosed using imaging techniques such as ultrasound or computed tomography. Cases that overlapped these four classifications mentioned above were labeled as mixed AKI. In this study, etiology of AKI was categorized as pre-renal AKI, HRS-AKI and other/mixed AKI, which included ATN-AKI and post-renal causes of AKI.

### ACLF

ACLF was defined according to European Foundation for the Study of Chronic Liver Failure (EF-CLIF) criteria and organ failures were defined according to the Chronic Liver Failure Consortium (CLIF-C) organ failure score.^22^ Renal dysfunction was defined by creatinine levels between 133-178 μmol/L and renal failure was defined by creatinine levels above 178 μmol/L).^9^ ACLF grades and scores were calculated using CLIF-C ACLF scores.^11^ Progression of ACLF was reported when there was an increase in the number of organ failures and/or an increase in the CLIF-C organ failure score.

### Statistical analysis

Baseline characteristics and data on disease history and severity were reported as mean and SD for normally distributed continuous variables. Not-normally distributed variables were presented as median and IQR. Binary categorical variables were compared with a Fisher’s exact test and a Kruskal Wallis test was used for multiple groups. Continuous variables were compared using the *t* test, and the Mann–Whitney *U* test was applied when the assumption of normality was violated. A *p* value <0.05 was considered statistically significant.

Univariate and multivariate logistic regression models were used to investigate the association between AKI characteristics and AKI outcomes, as well as ACLF development/progression. Potential risk factors, including well known precipitating events such as bacterial infection, gastrointestinal bleeding and toxic encephalopathy, were investigated in relation to specific types of organ failure in ACLF.^23^ Other known risk factors, including sex, age, comorbidities, model for end-stage liver disease (MELD) score, CLIF-C acute decompensation (AD) score, AD at hospitalization, and AKI characteristics were selected based on previous literature and their significance in univariate analysis. To estimate the cumulative incidence of death from the time of AKI diagnosis, a competing risk model was used,^24^ with liver transplantation considered a competing event. The cumulative incidence of death at 28 days, 90 days and 1 year, along with corresponding 95% CIs, were reported. To assess the association between risk factors and mortality at 28 days, 90 days and 1 year, three cause-specific regression models were employed.

Statistical analyses were performed with IBM SPSS Statistics (Statistical Package for the Social Sciences, version 29.0). Competing risk analysis was performed in R environment (R version 4.5.0)^25^ with mstate library.^26^ All research was conducted in accordance with both the Declarations of Helsinki and Istanbul; the study was approved by the ethics institutional committee of the LUMC and the requirement for informed consent was waived.

## Results

Data from 672 patients with cirrhosis and AKI were collected retrospectively. Baseline characteristics of the study cohort and characteristics regarding liver disease severity upon hospitalization are shown in Table 1. The median age at hospitalization was 60 years (IQR 53–67), and 71% of patients were male. The median MELD score at hospitalization was 23 (IQR 18–29). Alcohol-related liver disease was the most frequent etiology of cirrhosis, affecting 316 patients (47%), followed by viral hepatitis in 86 patients (13%). Upon hospitalization, 573 patients (85%) presented with AD, with a median CLIF-C AD score of 62 [54-69]. The type of decompensation was most frequently progressive grade 3 or refractory ascites in 288 patients (50%), followed by bacterial infections in 275 patients (48%).

**Table 1:** Baseline characteristics of 672 hospitalized patients with cirrhosis and AKI.

Baseline characteristics	Study cohort (N = 672) n (%) or median [IQR]
Study site	
LUMC	248 (37)
UKM	424 (63)
Sex (n = 667)	
Male	476 (71)
Female	191 (29)
Age at hospitalization	60 [53-67]
Comorbidities (n = 668)	
DM	229 (34)
COPD/asthma	57 (9)
Cardiovascular diseases	114 (17)
Etiology of cirrhosis	
Alcohol	316 (47)
Viral hepatitis	86 (13)
MASLD	68 (10)
PBC/PSC/AIH	41 (6)
Other∗	78 (12)
Combination	83 (12)
MELD-score at hospitalization	23 [18-29]
Acute decompensation	573 (85)
CLIF-C AD score at hospitalization	62 [54-69]
Type of decompensating events at hospitalization (n = 573)	
Ascites	288 (50)
SBP/bacterial infections	275 (48)
Overt HE	154 (27)
GI-bleed	60 (10)
Presence of ACLF at time of AKI diagnosis	406 (60)
ACLF grade (n = 406)	
Grade 1	215 (53)
Grade 2	96 (24)
Grade 3	93 (23)
Types of organ failures (n = 406)	
Renal	357 (88)
Liver	83 (20)
Circulatory	81 (20)
Coagulation	61 (15)
Respiratory	72 (18)
Brain	31 (8)
CLIF-C ACLF score	45 [40-53]
CLIF-C OF-score	9 [7-11]
Number of organ failures	1 [1-3]
Laboratory results at hospitalization	
Creatinin (mmol/L)	143 [99-220]
Bilirubin (mmol/L)	63 [33-186]
INR	1.6 [1.3-2.0]
Leukocytes (10∗^9^/L)	8.4 [5.7-12.6]
Sodium (mmol/L)	135 [130-139]

Data are presented as n (%) for categorical variables and median [IQR] for continuous variables. ∗Other: A1-antitrypsin deficiency or cryptogenic cirrhosis.

ACLF, acute-on-chronic liver failure; AIH, autoimmune hepatitis; AKI, acute kidney injury; CLIF-C AD, Chronic Liver Failure Consortium acute decompensation; GI bleed, gastrointestinal bleeding; HE, Hepatic Encephalopathy; LT, Liver Transplantation; MASLD, metabolic dysfunction-associated steatotic liver disease; MELD, model for end-stage liver disease; OF, organ failure; PBC, primary biliary cholangitis; PSC, primary sclerosing cholangitis; SBP, spontaneous bacterial peritonitis.

### AKI characteristics and renal outcomes

Renal outcomes are shown in Table 2. All included patients met the ICA criteria for AKI. AKI was present in 386 (57%) patients at hospitalization, whereas 286 patients (43%) developed AKI during hospitalization. Most patients had stage 1 AKI (367 patients, 55%), followed by stage 2 (88 patients, 13%) and stage 3 (216 patients, 32%) at the time of AKI diagnosis. The etiology of AKI varied: 32% pre-renal, 33% HRS-AKI, and 35% other/mixed. Albumin was administered to 451 patients (67%), and 263 patients (39%) received terlipressin. In 161 patients (24%), RRT was applied. A partial or complete response of AKI was observed in 355 patients (53%) by the end of hospitalization, while AKI progression or lack of response to therapy occurred in 317 patients (47%). AKI characteristics and renal outcomes for each center are presented separately in the supplementary materials (Table S1). Multivariate logistic regression models were estimated and revealed five independent risk factors for the combined endpoint no response/progression of AKI during hospitalization (Table 3). Male sex (odds ratio [OR] 1.66, 95% CI 1.08-2.53) MELD score (OR 1.06, 95% CI 1.03-1.09), AD at admission (OR 1.99, 95% CI 1.12-3.56), HRS-AKI and other/mixed were found to be significantly associated with AKI progression/no response (OR 2.58, 95% CI 1.60-4.15 and OR 2.97. 95% CI 1.81-4.88, respectively).

**Table 2:** AKI management and outcomes during hospitalization.

Baseline data	n (%) or median [IQR]
AKI present at hospitalization	386 (57)
AKI development during hospitalization	286 (43)
AKI stage at diagnosis	
Stage 1	367 (55)
Stage 2	88 (13)
Stage 3	217 (32)
AKI etiology	
HRS-AKI	223 (33)
Pre-renal	215 (32)
Other/mixed	234 (35)
Treatments	
Albumin treatment	451 (67)
Albumin and terlipressin treatment	263 (39)
Need for RRT	161 (24)
Outcomes	
AKI status after hospitalization	
Complete response	270 (40)
Partial response	85 (13)
No response	115 (17)
Progression	202 (30)

Data are presented as n (%) for categorical variables and median [IQR] for continuous variables. AKI, acute kidney injury; HRS-AKI, hepatorenal syndrome-acute kidney injury; RRT, renal replacement therapy.

**Table 3:** Univariate and multivariate logistic regression analysis for final AKI outcome.

Independent variables	Univariate		Multivariate	
	Progression/no improvement of AKI	*p* value	Progression/no improvement of AKI	*p* value
	OR (95% CI)		OR (95% CI)	
Sex				
Female	Reference		Reference	
Male	1.55 (1.10-2.18)	**0.012**	1.66 (1.08-2.53)	**0.020**
Age	0.97 (0.98-1.00)	0.112		
CLIF AD-score	1.02 (1.00-1.04)	**0.014**		
MELD-score at hospitalization	1.07 (1.04-1.09)	**<0.001**	1.06 (1.03-1.09)	**<0.001**
AD at admission				
No	Reference		Reference	
Yes	1.94 (1.24-3.04)	**0.004**	1.99 (1.12-3.56)	**0.020**
AKI etiology				
Pre-renal AKI	Reference		Reference	
HRS-AKI	2.38 (1.62-3.50)	**<0.001**	2.58 (1.60-4.15)	**<0.001**
Other/mixed	1.80 (1.23-2.64)	**0.002**	2.97 (1.81-4.88)	**<0.001**
AKI stage				
1	Reference		Reference	
2	1.26 (0.79-2.02)	0.332	1.3 (0.58-1.84)	0.919
3	2.20 (1.56-3.10)	**<0.001**	1.23 (0.76-1.99)	0.404

Univariate and multivariate logistic regression analyses were performed to identify independent predictors of progression/no improvement of AKI. Results are presented as odds ratios with 95% CIs, *p* values <0.05 were considered statistically significant. Values in bold indicate significant associations. ACLF, acute-on-chronic liver failure; AD, acute decompensation; AKI, acute kidney injury; CLIF-C AD, Chronic Liver Failure Consortium acute decompensation; HRS-AKI, hepatorenal syndrome-acute kidney injury; MELD, model for end-stage liver disease; OR, odds ratio.

### Outcomes of hospitalization

Outcomes related to hospitalization are described in Table 4. Median length of hospitalization was 14 days (IQR 7-26) and 370 patients (55%) were admitted to the intensive care unit. Seventy-seven patients (11%) received a liver transplant, including 30 patients during the index hospitalization. A total of 250 patients (37%) died during hospitalization.

**Table 4:** Outcomes of hospitalization in 672 hospitalized patients with cirrhosis and AKI.

Outcomes of hospital admission	n (%) or median [IQR]
Total days of hospitalization	14 [7-26]
ICU admission	370 (55)
ACLF development (n = 266)	101 (38)
Days between AKI and ACLF development	5 [2-13]
Types of organ failures (n = 101)	
Renal	72 (71)
Liver	47 (47)
Respiratory	48 (48)
Circulatory	41 (41)
Coagulation	27 (27)
Brain	19 (19)
Progression of ACLF (n = 406)	106 (26)
Types of organ failures (n = 106)	
Renal	96 (91)
Liver	69 (64)
Respiratory	66 (62)
Circulatory	46 (43)
Coagulation	35 (33)
Brain	47 (44)
LT in follow-up	77 (11)
LT during hospitalization	30 (39)
Death during hospitalization	250 (37)

Data are presented as n (%) for categorical variables and median [IQR] for continuous variables. ACLF, acute-on-chronic liver failure; ICU, intensive care unit; LT, liver transplantation.

### ACLF outcomes

**Fig. 1:**
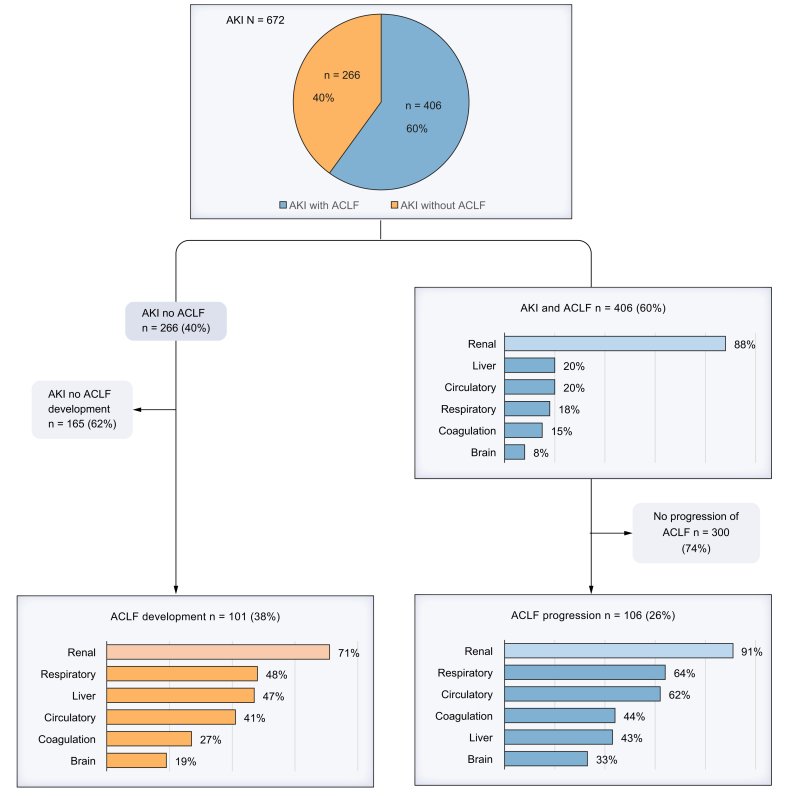
Clinical course of ACLF following AKI diagnosis. Flowchart illustrating the proportion of patients with ACLF at AKI diagnosis and those who showed ACLF progression, as well as the proportion of patients who developed ACLF after AKI diagnosis. Percentages indicate the distribution of organ failure types. ACLF, acute-on-chronic liver failure; AKI, acute kidney injury.

Outcomes regarding ACLF development and progression during hospitalization are shown in Fig. 1, Table 4 and S2. ACLF was diagnosed upon AKI diagnosis in 406 patients (60%), characterized by predominantly renal (88%) and liver (20%) failure, with median CLIF-C organ failure scores of 9 (IQR 7-11) (Table 1). Among patients without ACLF at the time of AKI diagnosis (n = 266), 101 (38%) developed ACLF during hospitalization with a median onset of 5 days [2-13]. Renal failure occurred in 72 patients (71%), whereas respiratory and liver failure were the most frequently occurring extrarenal organ failures (48% and 47% in 48 and 47 patients, respectively). Among 406 patients with ACLF at the time of AKI diagnosis (60%), 106 (26%) showed progression of ACLF during hospitalization, with predominantly renal, respiratory and circulatory failure (91%, 64% and 62%, respectively).

In multivariate logistic regression, patients with HRS-AKI or other/mixed AKI had a significantly higher risk of ACLF development compared to those with pre-renal AKI (OR 9.67, 95% CI 3.96-23.57 and OR 4.98, 95% CI 1.78-12.95, respectively), while higher MELD scores were also associated with increased risk (OR 1.18, 95% CI 1.09-1.27) (Table 5). In patients with ACLF at the time of diagnosis of AKI, history of cardiovascular disease (OR 3.11, 95% CI 1.47-6.58), MELD score (OR 1.09, 95% CI 1.05-1.14), AKI stage 2 (OR 2.35, 95% CI 1.03-5.36) and HRS-AKI (OR 2.31, 95% CI 1.08-4.95) were independently associated with ACLF progression. Univariate logistic regression analysis was performed separately for each type of organ failure (Table S3), with comorbidities, AKI stage, and AKI etiology included as potentially associated risk factors. HRS-AKI was associated with higher odds of liver failure, whereas AKI stage and etiology were not consistently linked to renal failure in either ACLF development or progression. Overall, most comorbidities and AKI characteristics showed limited predictive value for specific types of organ failure. In the LUMC study cohort, data on cumulative albumin dosages were available (Table S4). No significant associations were observed between albumin dose and specific organ failure types in ACLF development and progression, except for coagulation failure, which was less frequent in patients receiving >300 g of albumin (OR 0.41, 95% CI 0.18-0.93).

**Table 5:** Univariate and multivariate logistic regression analysis on ACLF development and ACLF progression.

Independent variables	Univariate analysis		Multivariate analysis		Univariate analysis		Multivariate analysis	
	Development of ACLF	*p* value	Development of ACLF	*p* value	Progression of ACLF	*p* value	Progression of ACLF	*p* value
	OR (95% CI)		OR (95% CI)		OR (95% CI)		OR (95% CI)	
Sex								
Male	Reference		Reference		Reference		Reference	
Female	0.63 (0.36-1.11)	0.108	0.63 (0.27-1.46)	0.279	1.36 (0.84-2.21)	0.212	1.55 (0.86-2.79)	0.144
Age	0.98 (0.98-1.00)	0.084	1.00 (0.97-1.04)	0.825	0.99 (0.97-1.01)	0.145	1.00 (0.97-1.03)	0.866
Comorbidities								
Diabetes mellitus	0.64 (0.39-1.06)	0.080			0.66 (0.42-1.03)	0.067		
COPD	1.57 (0.69-3.58)	0.288			0.77 (0.32-1.83)	0.549		
Cardiovascular disease	0.89 (0.49-1.59)	0.681			2.28 (1.23-4.20)	**0.009**	3.11 (1.47-6.58)	**0.003**
MELD-score	1.15 (1.08-1.22)	**<0.001**	1.18 (1.09-1.27)	**<0.001**	1.08 (1.04-1.12)	**<0.001**	1.09 (1.05-1.14)	**<0.001**
CLIF AD-score	1.02 (0.99-1.02)	0.215			1.04 (1.02-1.07)	**<0.001**		
AD at admission								
Yes	2.26 (1.27-4.01)	**0.006**	0.40 (0.03-5.46)	0.493	1.33 (0.65-2.70)	0.433		
No	Reference		Reference		Reference			
AKI stage								
1	Reference				Reference		Reference	
2	1.38 (0.65-2.92)	0.737			2.80 (1.42-5.54)	**0.003**	2.35 (1.03-5.36)	**0.042**
3	3.11 (0.88-10.94)	0.078			1.71 (1.01-2.87)	**0.042**	0.85 (0.43-1.68)	0.641
AKI etiology								
Pre-renal	Reference		Reference		Reference		Reference	
HRS-AKI	8.40 (4.25-16.61)	**<0.001**	9.67 (3.96-23.57)	**<0.001**	2.78 (1.53-5.05)	**<0.001**	2.31 (1.08-4.95)	**0.031**
Other/mixed	2.01 (1.04-3.87)	**0.037**	4.98 (1.78-12.95)	**0.002**	1.51 (0.8-2.82)	0.192	2.17 (0.99-4.73)	0.052

**Precipitating events**								
Bacterial infection								
Yes	2.33 (1.34-4.05)	**0.003**	1.02 (0.46-2.25)	0.960	1.32 (0.83-2.10)	0.245		
No	Reference		Reference		Reference			
GI-bleed								
Yes	0.77 (0.30-2.00)	0.595			0.74 (0.34-1.63)	0.458		
No	Reference				Reference			
HE								
Yes	1.19 (0.65-2.18)	0.581			1.62 (0.98-2.70)	0.062		
No	Reference				Reference			

Univariate and multivariate logistic regression analyses were performed to identify independent predictors of ACLF development and progression. Results are presented as odds ratios with 95% CIs, *p* values <0.05 were considered statistically significant. ACLF, acute-on-chronic liver failure; AD, acute decompensation; AKI, acute kidney injury; CLIF-C AD, Chronic Liver Failure Consortium acute decompensation; GI-bleed, gastrointestinal bleeding; HE, Hepatic Encephalopathy; MELD, model for end-stage liver disease; OR, odds ratio.

### Survival outcomes and follow-up

**Fig. 2:**
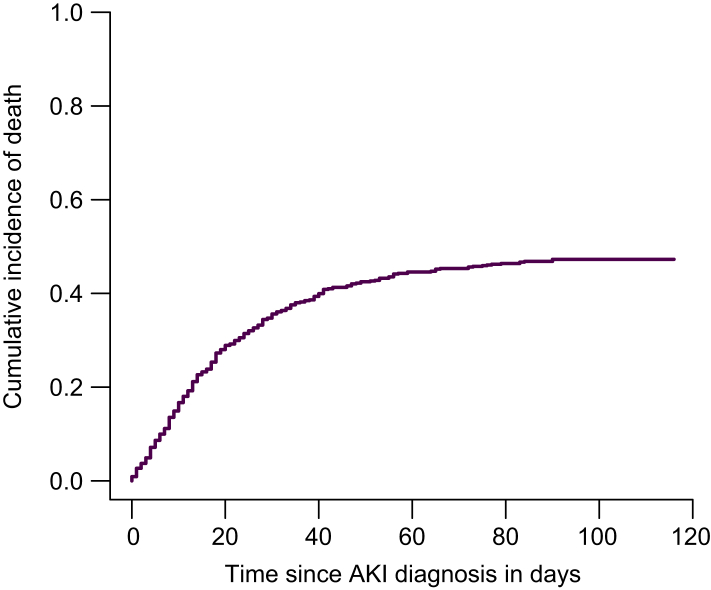
Cumulative incidence of death following AKI diagnosis. The x-axis indicates time since AKI diagnosis in days, the y-axis shows the cumulative proportion of patients who died during follow-up. Liver transplantation was treated as a competing risk. AKI, acute kidney injury.

Median follow-up was 52 days [15-365]. The cumulative incidence of death was 34% at 28 days (95% CI 32-38), 47% at 90 days (95% CI 44-51), and 53% at 1 year after AKI diagnosis (95% CI 49-56) (Fig. 2). Cause-specific regression identified higher MELD scores, AD at admission and patients who showed no response/AKI progression as independent risk factors for 28-day, 90-day, and 1-year mortality (Tables 6, S5 and S6, respectively). To evaluate the impact of the 2015 implementation of the new ICA AKI definitions,^5^ patient characteristics and mortality rates among patients admitted with AKI before and after this time point were compared. Patients hospitalized after 2015 were slightly older (62 *vs*. 59 years, *p <*0.001) and had a higher proportion of HRS-AKI (39% *vs.* 25%, *p <*0.001). Patients diagnosed after 2015 had a significantly higher 28-day mortality rate compared to those hospitalized before 2015 (37% *vs.* 29%, *p =* 0.031). No significant differences were observed in longer-term mortality rates (90-day and 1-year) between the two periods (Table S7).

**Table 6:** Univariate and multivariate cause specific regression model for 28-day mortality.

Independent variables	Univariate		Multivariate	
	Mortality	*p* value	Mortality	*p* value
	HR (95% CI)		HR (95% CI)	
Sex				
Male	Reference		Reference	
Female	0.77 (0.57-1.04)	0.088	1.12 (0.80-1.56)	0.511
Age	1.00 (0.99-1.01)	0.721	1.01 (0.99-1.02)	0.476
CLIF-C AD score	1.05 (1.03-1.06)	**<0.001**		
MELD-score at admission	1.07 (1.05-1.09)	**<0.001**	1.05 (1.02-1.07)	**<0.001**
AD at admission				
No	Reference		Reference	
Yes	2.78 (1.67-4.63)	**<0.001**	2.49 (1.38-4.50)	**0.003**
ACLF status				
No ACLF	Reference		Reference	
ACLF at time of AKI diagnosis	3.82 (2.42-6.02)	**<0.001**	1.44 (0.77-2.69)	0.251
ACLF development	4.39 (2.62-7.34)	**<0.001**	1.49 (0.79-2.82)	0.215
AKI etiology				
Pre-renal AKI	Reference		Reference	
HRS-AKI	1.60 (1.16-2.21)	**0.005**	0.80 (0.54-1.17)	0.247
Other/mixed	1.23 (0.87-1.72)	**0.238**	1.02 (0.69-1.50)	0.990
AKI response end of admission				
Partial/complete response	Reference		Reference	
No response/progression	5.70 (4.16-7.80)	**<0.001**	4.67 (3.16-6.91)	**<0.001**
AKI stage				
1	Reference		Reference	
2	1.63 (1.11-2.39)	**0.013**	1.46 (0.94-2.26)	0.092
3	1.78 (1.34-2.36)	**<0.001**	1.02 (0.69-1.50)	0.935

Univariate and multivariate cause specific regression analyses were performed to identify independent predictors of 28-day mortality. Results are presented as Hazard ratios with 95% confidence intervals, *p* values <0.05 were considered statistically significant. ACLF, acute-on-chronic liver failure; AD, acute decompensation; AKI, acute kidney injury; CLIF-C AD score, Chronic Liver Failure Consortium acute decompensation; HR, hazard ratio; HRS-AKI, hepatorenal syndrome-acute kidney injury; MELD, model for end-stage liver disease.

## Discussion

AKI is a frequent and severe complication in patients with cirrhosis. While ACLF is defined as a dynamic syndrome characterized by multi-organ failure and high short-term mortality, the timing and patterns of ACLF development or progression in the context of AKI have not been systematically described. In this study, we investigated the clinical course of AKI in hospitalized patients with cirrhosis, specifically distinguishing between renal and extrarenal organ failures in those diagnosed with ACLF. We observed that ACLF frequently developed within 28 days following AKI diagnosis, with respiratory, liver, and circulatory failure being the most common extrarenal organ failures.

Diagnosing AKI in patients with cirrhosis according to its specific etiology can be challenging due to overlapping clinical presentations, and a thorough understanding of the current diagnostic criteria is essential for effective clinical management. In this study, HRS-AKI (33%) was the most frequent etiology, followed by pre-renal AKI (32%). Notably, in our study, patients diagnosed with HRS-AKI in combination with other types of AKI were categorized under the mAKI group. In comparison, Ma *et al.* (2024)^16^ reported HRS-AKI in approximately 26% of the AKI cases in patients with cirrhosis, while Patidar *et al.*^13^ found an even lower incidence of 17%. Differences in infection rates across cohorts may partly explain these variations, as patients with spontaneous bacterial peritonitis or other bacterial infections are prone to develop HRS-AKI due to increased levels of vasodilatory mediators, leading to a decrease in renal blood flow.^7^^,^^27^ In our study, spontaneous bacterial peritonitis or bacterial infections were found in 44% of patients. In Ma *et al.*, infections occurred in 56% of patients at the time of AKI diagnosis, whereas in Patidar *et al.* infections occurred in 30%.

In terms of AKI treatment, albumin was administered to 67% of patients, similar to the study by Patidar *et al.*, where albumin was prescribed in 67% of all cases.^21^ In one of the two study cohorts, cumulative albumin dosages were available. The median cumulative dose of albumin for the treatment of HRS-AKI administered in this cohort was higher (385 g) than that reported in studies by Wong *et al.* and Martin-Ilahi *et al.*, in which patients with cirrhosis and AKI treated with terlipressin and albumin for HRS-AKI received approximately 199 g of albumin over a median of 5 days and 190 g of albumin over a median of 7 days, respectively.^28^^,^^29^ Importantly, in our study, in which we closely adhered to the valid guidelines for AKI treatment in cirrhosis at that time, higher cumulative dosages of albumin were not independently associated with the development of any type of organ failure in ACLF. It may be hypothesized that prolonged treatment duration and the higher frequency of HRS-AKI explain the observed differences in cumulative albumin dosages as compared to the previously mentioned studies.

Response rates to treatment, defined as partial or complete response of AKI, differed between etiologies and stages. In this study, partial or complete response was observed in 53% of patients. By comparison, Ma *et al.* reported a response rate of 80% and Patidar *et al.* observed partial/full response of AKI in 75% of cases.^13^^,^^16^

The higher severity of AKI stages observed in our study, with a larger proportion of patients presenting with stage 3 AKI at diagnosis (32% *vs.* 17% and 20% in the other studies), may account for the lower response rates observed.

During hospitalization, ACLF was diagnosed in 507 patients (75%), of whom 406 patients were diagnosed with ACLF upon AKI diagnosis. In these patients, renal failure was the most frequent type of organ failure, followed by liver and circulatory failure. Given that ACLF criteria for renal dysfunction are determined on serum creatinine thresholds (133-168 μmol/L for dysfunction and >177 μmol/L for failure), while AKI diagnosis follows ICA criteria, only patients with more severe AKI met ACLF renal failure criteria. This is supported by recent reports that the kidneys are differentially affected in patients with ACLF when compared to patients with decompensated cirrhosis, as they are more likely to have evidence of structural and tubular damage.^14^^,^^30^^,^^31^ These findings are consistent with the previous study of Patidar *et al.*, in which 59% of patients had ACLF at AKI onset with a similar distribution of organ failures.^13^ Huelin *et al.* reported comparable ACLF rates at admission in patients with AKI (70%), although the pattern of organ failures varied slightly according to AKI stage.^18^ Notably, among patients who developed ACLF during hospitalization, respiratory failure – most frequently due to fluid overload – emerged as the second most prevalent organ failure following renal failure. This is in contrast with the findings of the CANONIC study,^9^ in which renal failure was the most prevalent organ failure in unselected patients hospitalized with acute decompensation of cirrhosis and ACLF, followed by liver failure, with respiratory failure being the least common type of organ failure. Furthermore, patients who developed ACLF after AKI diagnosis showed higher incidences of circulatory failure in this study (41%) than in the CANONIC study (9%), indicating a higher susceptibility to circulatory instability. It is important to note that in this study, terlipressin was administered exclusively for the treatment of HRS-AKI and not used as a vasoconstrictor for managing circulatory failure in ACLF.

In the present study, high mortality rates were observed among patients with cirrhosis and AKI, with 28-day, 90-day and 1-year mortality rates of 34%, 47% and 53%, respectively, and an in-hospital mortality rate of 37%. The 90-day mortality of 47% lies at the upper range of previously reported outcomes: Wong *et al.*^28^ reported mortality rates of 45-51% in patients with HRS-AKI, compared with 37% in Patidar *et al.*,^21^ and 32% in Ma *et al.*^16^ Differences in liver transplantation rates are unlikely to explain this variation, as 11% of patients in our cohort underwent transplantation (including 30 during the same hospitalization), comparable to the rates observed by Ma (7%) and Patidar (8%). A more plausible explanation might be the higher baseline disease severity and differences in AKI etiology. The present cohort included a higher proportion of HRS-AKI, consistent with the observed proportion by Wong *et al.*, whereas fewer HRS-AKI cases were included in the studies by Ma *et al.* and Patidar *et al.* Moreover, in our study, some patients with HRS-AKI also had additional AKI etiologies and were classified as other/mixed AKI. This overlap may have contributed to the higher observed mortality, although other factors, such as inclusion criteria and management strategies may also have contributed to the observed discrepancy. In the multivariable cause-specific regression model, a higher MELD score, the presence of AD at admission, and AKI non-response or progression were independently associated with 28-day, 90-day, and 1-year mortality. These results highlight the need for comprehensive risk stratification and targeted interventions in patients at high risk of mortality.

A key strength of this study is that the development and clinical course of ACLF was followed very precisely, differentiating between the presence of ACLF at the time of AKI diagnosis, and subsequently at development and progression. In addition, the course of AKI and survival was closely monitored up to 1 year, providing accurate results regarding AKI and survival outcomes. Finally, this study includes only unique AKI episodes per patient, whereas other literature includes multiple AKI episodes per patient.^16^ Several limitations should be noted. There were some differences in baseline characteristics and AKI etiology between the two study cohorts, although AKI response rates were similar. Respiratory failure occurred in both cohorts as the most frequent extrarenal organ failure following renal failure in patients with ACLF development or progression, highlighting a crucial and consistent aspect of the clinical trajectory in these severely ill patients. The fact that both sites are tertiary referral hospitals with liver transplant facilities in different countries may introduce a potential selection bias and limit the generalizability of our findings. We also recognize that confounding factors inherent to the retrospective study design may have influenced outcomes. In addition, uNGAL (urinary neutrophil gelatinase-associated lipocalin) was not integrated into routine diagnostic protocols within this retrospective study, potentially leading to the misclassification of AKI etiology, as uNGAL has been demonstrated to be a suitable biomarker in the differential diagnosis between ATN-AKI and HRS-AKI in previous studies.^16^^,^^20^^,^^32^ Finally, ATN was not classified as a separate category in our study, despite previous literature demonstrating that ATN-AKI is associated with worse outcomes.^16^^,^^21^^,^^32^

In conclusion, patients with cirrhosis and AKI face very high short-term mortality and are at substantial risk of ACLF development and progression, with respiratory failure being the most frequent extrarenal organ failure. Developing novel therapeutic approaches, particularly for non-responders, and conducting prospective studies to refine risk stratification models and elucidate the underlying mechanisms driving organ failure are essential to improve outcomes in this population.

## Abbreviations

ACLF, acute-on-chronic liver failure; AD, acute decompensation; AKI, acute kidney injury; ATN, acute tubular necrosis; CLIF-C, Chronic Liver Failure Consortium HRS-AKI, hepatorenal syndrome-acute kidney injury; ICA, International Club of Ascites; MELD, model for end-stage liver disease; OR, odds ratio; RRT, renal replacement therapy.

## Authors contributions

SF, MSM, JB, JN, JT and MC conceptualized and designed the study. SF and MSM contributed to the acquisition of data, analysis and interpretation of the data. SF drafted the manuscript. MF performed competing risk analysis and provided advises about the statistical analysis, interpretation of the data and critical revision of the manuscript for important intellectual content. ECA, JH, MP, MJB, FEU, LW, SZ, JC, and KHP contributed to acquisition of the data and critical revision of the manuscript for important intellectual content. MSM, AK, SA, JB, JN, DvW, AJvZ participated in the critical revision of the paper for important intellectual content. MC and JT participated in the interpretation of the data, drafting the manuscript, critical revision of the manuscript for important intellectual content, and study supervision.

## Data availability

The datasets used and analyzed during the current study are available from the corresponding author upon reasonable request.

## Declaration of AI and AI-assisted technologies in the writing process

During the preparation of this work the author(s) used ChatGPT and Gemini in order to ensure adherence to the British English standards and improve overall readability. After using this tool, the author(s) reviewed and edited the content as needed and take(s) full responsibility for the content of the publication.

## Financial support

MC has received funding from the European Union's Horizon 2020 research and innovation program for MICROB-PREDICT (project ID 825694), Health Holland PPI and Gastrostart. JT is supported by grants from Deutsche Forschungsgemeinschaft (SFB TRR57 to P18), European Union’s Horizon 2020 Research and Innovation Programme (Galaxy, No. 668031 and MICROB-PREDICT, No. 825694) and Societal Challenges - Health, Demographic Change and Wellbeing (No. 731875), and Cellex Foundation (PREDICT). SZ has received consultancy and/or speaker fees from AbbVie, Boehringer Ingelheim, Gilead, GSK, Ipsen, Madrigal, Novo Nordisk, and MSD/Merck. SF has received funding from the European Union's Horizon 2020 research and innovation program for MICROB-PREDICT (project ID 825694). The funders had no role in study design, data collection and analysis, decision to publish, or preparation of the manuscript.

## Conflict of interest

No conflict of interest to disclose.

Please refer to the accompanying ICMJE disclosure forms for further details.
