# Supplementary material for: Development and patterns of acute-on-chronic liver failure in patients with cirrhosis and acute kidney injury
Source: JHEP Rep. 2026 Jan 12;8(3):101734. doi: 10.1016/j.jhepr.2026.101734 (PMC12926641; doi:10.1016/j.jhepr.2026.101734)
Supplement: Multimedia component 1 [file mmc1.pdf]

Development and patterns of acute-on-chronic liver failure in patients with cirrhosis and acute kidney injury

Susan Fischer, Martin Sebastian McCoy, Marta Fiocco, Annarein Kerbert, Eduardo Cervantes-Alvarez, Jan Hähner, Michael Praktiknjo, Maximilian Joseph Brol, Frank Erhard Uschner, Lena Wolters, Stefan Zeuzem, Josune Cabello , Kai-Henrik Peiffer, Jeetindra Balak, Sesmu Arbous, Jeroen Nieuwenhuizen, David van Westerloo, Anton Jan van Zonneveld, Jonel Trebicka, Minneke Coenraad

Table of contents

Table S1 ……..…………………………………………………..……………………….…2

Table S2 …………..…………………………………………………………………….....3

Table S3 ………………………………………………………………….……...……........4

Table S4……………………………………..…………………….…...………….. 5

Table S5 ………………………………..………………………………………………....6

Table S6 ……………………………..……………………………………………..………7

Table S7……………………………………………………….…………………..……….…7

Table S1. AKI characteristics and hospital outcomes of both study cohorts

AKI, ACLF and hospital outcomes	Study cohort 1 LUMC (n=248)	Study cohort 2 UKM (n=424)	Both cohorts (n=672)
AKI present at time admission	189 (76)	197 (47)	386 (57)
AKI development during hospitalization	59 (24)	227 (53)	286 (43)
AKI stage at diagnosis			
Stage 1	146 (59)	221 (52)	367 (55)
Stage 2	62 (25)	26 (6)	88 (13)
Stage 3	40 (16)	176 (42)	216 (32)
AKI etiology			
HRS-AKI	110 (44)	112 (26)	223 (33)
Pre-renal	88 (36)	127 (30)	215 (32)
Other/mixed AKI	50 (20)	184 (44)	234(35)
AKI treatments			
Albumin treatment	212 (86)	240 (57)	451 (67)
Terlipressin	151 (61)	138 (33)	263 (39)
Need for RRT	84 (34)	106 (25)	161 (24)
AKI status after hospitalization			
Complete response	99 (40)	171 (40)	270 (40)
Partial response	23 (9)	62 (15)	85 (13)
No response	33 (13)	81 (19)	115 (17)
Progression	93 (38)	109 (26)	202 (30)
ACLF outcomes			
Any ACLF phenotype at/after AKI diagnosis	174 (70)	333 (79)	507 (75)
Hospital outcomes<br>Days of admission (median ◦ IQR)	14 [7-26]	13 [7-24]	14 [7-26]
ICU admission	141 (57)	229 (54)	370 (55)
LT in the follow-up period	54 (22)	23 (5)	77 (11)
Mortality			
28-day mortality	108 (41)	122 (29)	230 (34)
90-day mortality	148 (58)	171 (40)	319 (47)
1-year mortality	162 (65)	192 (45)	354 (53)
In-hospital mortality	114 (46)	136 (32)	250 (37)

Data are presented as number (%) for categorical variables and median [IQR] for continuous variables. Abbreviations: ACLF, acute-on-chronic liver failure; AKI, acute kidney injury; ICU, intensive care unit; IQR, interquartile range; LT, liver transplantation; RRT, renal replacement therapy

*missing data n=2

Table S2: ACLF characteristics of both study cohorts

**Baseline characteristics**	**Study cohort 1 LUMC (n=248)**<br><br>**N (%), median [IQR]**	**Study cohort 2 UKM (n=424)**<br><br>**N (%), median [IQR]**	**Both cohorts (n=672)**<br><br>**N (%), median [IQR]**
Presence of ACLF at time of AKI diagnosis	101 (41)	305 (72)	406 (60)
ACLF grade			
Grade 1	47 (46)	168 (55)	215 (53)
Grade 2	24 (24)	72 (24)	96 (24)
Grade 3	30 (30)	63 (21)	93(23)
Types of organ failures (n=101)			
Renal	71 (70)	286 (94)	357 (88)
Liver	45 (45)	38 (13)	83 (20)
Circulatory	32 (32)	49 (16)	81 (20)
Coagulation	24 (24)	37 (12)	61 (15)
Respiratory	23 (23)	49 (12)	72 (18)
Brain	20 (20)	11 (4)	31 (8)
OF-score (median ◦ IQR)	10 [9-13]	8 [6-9]	9 [7-11]
ACLF score (median ◦ IQR)	51 [44-61]	43 [37-50]	45 [40-53]
Number of organ failures (median ◦ IQR)	2 [1-3]	1 [1-2]	1 [1-3]
ACLF development	73 (50)	28 (23)	101
ACLF grade			
Grade 1	7 (10)	18 (64)	25 (25)
Grade 2	24 (35)	6 (21)	30 (30)
Grade 3	38 (55)	4 (14)	42 (41)
Types of organ failures (n=101)			
Renal	48 (67)	24 (86)	72 (71)
Liver	44 (60)	3 (11)	47 (47)
Respiratory	43 (59)	5 (18)	48 (48)
Circulatory	40 (55)	1 (4)	41 (41)
Coagulation	19 (26)	8 (29)	27 (27)
Brain	18 (25)	1 (4)	19 (19)
Progression of ACLF	57 (56)	49 (16)	106 (26)
ACLF grade at time of progression			
Grade 2	10 (18)	19 (39)	29 (27)
Grade 3	47 (82)	30 (61)	77 (73)
Types of organ failures			
Renal	48 (84)	48 (98)	96 (91)
Respiratory	39 (68)	29 (59)	69 (64)
Circulatory	39 (68)	27 (55)	66 (62)
Liver	37 (65)	9 (18)	46 (43)
Brain	33 (58)	2 (4)	35 (33)
Coagulation	21 (37)	26 (53)	47 (44)

Data are presented as number (%) for categorical variables and median [IQR] for continuous variables.
Abbreviations: ACLF, acute-on-chronic liver failure; OF, organ failure; IQR, interquartile range; GI bleed, gastrointestinal bleeding; COPD, chronic obstructive pulmonary disease

Table S3: Univariate logistic regression analysis on types of ACLF organ failures.

Independent variables	ACLF development Organ Failure type<br><br>OR (95% CI)	p-value	ACLF progression Organ Failure type<br><br>OR (95% CI)	p-value
	**ACLF development Circulatory Failure**		**ACLF progression Circulatory Failure**	
Comorbidities				
Diabetes mellitus	0.32 (0.14-0.76)	**0.009**	0.64 (0.29-1.41)	0.270
COPD	1.05 (0.31-3.58)	0.936	n/a	0.999
Cardiovascular disease	2.32 (0.90-5.98)	0.081	1.27 (0.46-3.47)	0.643
AKI stage				
1	Reference		Reference	
2	0.71 (0.22-2.32)	0.714	0.96 (0.31-2.98)	0.944
3	0.21 (0.03-1.86)	0.214	1.64 (0.64-4.19)	0.299
AKI etiology				
Pre-renal AKI	Reference		Reference	
HRS-AKI	1.40 (0.48-4.06)	0.536	2.05 (0.71-5.91)	0.185
Other/mixed AKI	1.63 (0.51-5.21)	0.414	2.13 (0.68-6.66)	0.193
	**ACLF development Respiratory Failure**		**ACLF progression Respiratory Failure**	
Comorbidities				
Diabetes mellitus	0.67 (0.30-1.49)	0.327	0.42 (0.18-0.94)	**0.036**
COPD	1.64 (0.48-5.56)	0.428	3.43 (0.40-29.62)	0.263
Cardiovascular disease	2.56 (0.97-6.74)	0.057	1.09 (0.40-3.00)	0.866
AKI stage				
1	Reference		Reference	
2	1.55 (0.49-4.88)	0.454	1.40 (0.44-4.44)	0.944
3	0.86 (0.18-4.15)	0.863	1.64 (0.64-4.19)	0.299
AKI etiology				
Pre-renal AKI	Reference		Reference	
HRS-AKI	1.69 (0.60-4.77)	0.322	1.66 (0.58-4.78)	0.350
Other/mixed AKI	1.52 (0.48-4.76)	0.475	1.96 (0.62-6.20)	0.250
	**ACLF development Renal Failure**		**ACLF progression Renal Failure**	
Comorbidities				
Diabetes mellitus	1.84 (0.74-4.60)	0.192	2.00 (0.56-7.10)	0.283
COPD	0.74 (0.20-2.68)	0.645	n/a	0.999
Cardiovascular disease	3.15 (0.85-11.58)	0.085	0.29 (0.08-1.02)	0.054
AKI stage				
1	Reference		Reference	
2	0.64 (0.19-2.13)	0.468	1.44 (0.30-6.83)	0.647
3	0.89 (0.16-4.94)	0.894	4.17 (0.92-18.94)	0.065
AKI etiology				
Pre-renal AKI	Reference		Reference	
HRS-AKI	0.68 (0.21-2.19)	0.522	0.94 (0.17-5.12)	0.944
Other/mixed AKI	0.98 (0.26-3.66)	0.979	0.92 (0.15-5.50)	0.920
	**ACLF development Liver Failure**		**ACLF progression Liver Failure**	
Comorbidities				
Diabetes mellitus	0.37 (0.16-0.83)	**0.017**	0.41 (0.19-0.91)	**0.028**
COPD	0.54 (0.15-1.91)	0.334	0.52 (0.10-2.82)	0.449
Cardiovascular disease	0.85 (0.33-2.18)	0.738	1.30 (0.50-3.39)	0.593

AKI stage				
1	Reference		Reference	
2	0.79 (0.25-2.48)	0.684	3.12 (0.96-10.15)	0.059
3	0.42 (0.08-2.30)	0.317	0.77 (0.30-1.96)	0.577
AKI etiology				
Pre-renal AKI	Reference		Reference	
HRS-AKI	4.57 (1.15-14.42)	**0.010**	9.86 (2.07-46.91)	**0.004**
Other/mixed AKI	2.26 (0.65-7.86)	0.200	7.16 (1.43-35.78)	0.017
	**ACLF development Coagulation Failure**		**ACLF progression Coagulation Failure**	
Comorbidities				
Diabetes mellitus	0.59 (0.23-1.48)	0.259	1.09 (0.51-2.34)	0.822
COPD	0.22 (0.03-1.79)	0.157	0.63 (0.13-2.94)	0.552
Cardiovascular disease	1.66 (0.61-4.51)	0.324	0.93 (0.36-2.43)	0.886
AKI stage				
1	Reference		Reference	
2	0.72 (0.18-2.82)	0.636	1.56 (0.50-4.85)	0.446
3	1.06 (0.19-5.84)	0.952	1.422 (0.57-3.56)	0.452
AKI etiology				
Pre-renal AKI	Reference		Reference	
HRS-AKI	1.21 (0.37-3.93)	0.750	1.20 (0.42-3.41)	0.737
Other/mixed AKI	1.22 (0.34-4.44)	0.764	1.88 (0.61-5.83)	0.274
	**ACLF development Brain Failure**		**ACLF progression Brain Failure**	
Comorbidities				
Diabetes mellitus	0.98 (0.36-2.68)	0.963	0.32 (0.13-0.75)	**0.009**
COPD	0.36 (0.04-2.96)	0.341	0.84 (0.15-4.55)	0.837
Cardiovascular disease	0.68 (0.40-4.00)	0.683	2.97 (1.11-7.91)	0.030
AKI stage				
1	Reference		Reference	
2	0.29 (0.04-2.34)	0.242	3.43 (1.03-11.41)	**0.045**
3	0.62 (0.07-5.48)	0.665	1.09 (0.39-3.07)	0.873
AKI etiology				
Pre-renal AKI	Reference		Reference	
HRS-AKI	2.32 (0.46-11.62)	0.307	3.31 (0.81-18.84)	0.090
Other/mixed AKI	3.02 (0.56-16.33)	0.199	7.16 (1.43-35.78)	**0.017**

Univariate and multivariate logistic regression analyses were performed to identify independent predictors of all types of organ failure in patients with ACLF development and progression. Results are presented as odds ratios with 95% confidence intervals, p-values <0.05 were considered statistically significant. Abbreviations: AKI, acute kidney injury; ACLF, acute-on-chronic liver failure; HRS-AKI, hepatorenal syndrome-acute kidney injury; CI, confidence interval; OR, odds ratio.

Table S4: Cumulative dosages of albumin on all types of organ failures in ACLF (LUMC cohort).

	**Respiratory failure OR (95% CI)**	**p-value**	**Circulatory failure OR (95% CI)**	**p-value**	**Renal failure OR (95% CI)**	**p-value**
Cum. albumin dose						
< 300 grams	Reference		Reference		Reference	
> 300 grams	0.63 (0.28-1.43)	0.274	0.49 (0.22-1.11)	0.087	0.94 (0.39-2.26)	0.886
	**Liver failure OR (95% CI)**	**p-value**	**Brain failure OR (95% CI)**	**p-value**	**Coagulation failure OR (95% CI)**	**p-value**
Cum. albumin dose						
< 300 grams	Reference		Reference		Reference	
> 300 grams	0.91 (0.41-2.02)	0.811	0.81 (0.37-1.76)	0.595	0.41 (0.18-0.93)	**0.032**

Univariate logistic regression analyses were performed to assess the association between cumulative albumin dose and the development of each type of organ failure in ACLF. Results are presented as odds ratios with 95% confidence intervals, p-values <0.05 were considered statistically significant. Abbreviations: CI, confidence interval; OR, odds ratio.

Table S5. Univariate and multivariate cause specific regression model for 90-day mortality

**Independent variables**	**Mortality** **Univariate** HR (95% CI)	p-value	**Mortality** **Multivariate** HR (95% CI)	p-value
Sex				
Male	Reference		Reference	
Female	0.78 (0.61-1.01)	0.055	1.11 (0.84-1.48)	0.460
Age	1.00 (0.99-1.01)	0.442	1.01 (0.99-1.02)	0.413
CLIF-C AD score	1.04 (1.02-1.05)	**<0.001**		
MELD-score at admission	1.06 (1.04-1.08)	**<0.001**	1.05 (1.03-1.07)	**<0.001**
AD at admission				
No	Reference		Reference	
Yes	2.29 (1.56-3.38)	**<0.001**	2.19 (1.39-3.44)	**<0.001**
ACLF status				
No ACLF	Reference		Reference	
ACLF at time of AKI diagnosis	2.26 (1.65-3.11)	**<0.001**	0.87 (0.55-1.38)	0.560
ACLF development	3.49 (2.41-5.07)	**<0.001**	1.27 (0.80-2.01)	0.306
AKI etiology				
Pre-renal AKI	Reference		Reference	
HRS-AKI	1.62 (1.24-2.13)	**<0.001**	1.00 (0.72-1.38)	0.987
Other/mixed	1.10 (0.83-1.46)	**0.521**	1.04 (0.74-1.47)	0.815
AKI response end of admission				
Partial/Complete response	Reference		Reference	
No response/Progression	4.17 (3.28-5.31)	**<0.001**	3.89 (2.86-5.29)	**<0.001**
AKI stage				
1	Reference		Reference	
2	1.28 (0.92-1.79)	0.149	1.39 (0.95-1.34)	0.087
3	1.35 (1.06-1.72)	**0.015**	0.95 (0.68-1.34)	0.783

Univariate and multivariate cause specific regression analyses were performed to identify independent predictors of 90-day mortality. Results are presented as hazard ratios with 95% confidence intervals, p-values <0.05 were considered statistically significant. Abbreviations: CLIF-C AD score, Chronic Liver Failure Consortium Acute Decompensation score; AKI, acute kidney injury; AD, acute decompensation; MELD, Model For End-Stage Liver Disease; ACLF, acute-on-chronic liver failure; HRS-AKI, hepatorenal syndrome-acute kidney injury; CI, confidence interval; OR, odds ratio.

Table S6. Univariate and multivariate cause specific regression model for 1 year mortality

Independent variables	Mortality<br><br>**Univariate**<br><br>HR (95% CI)	p-value	Mortality<br><br>**Multivariate**<br><br>HR (95% CI)	p-value
Sex				
Male	Reference		Reference	
Female	0.81 (0.64-1.02)	0.074	1.10 (0.84-1.44)	0.503
Age	1.00 (0.99-1.01)	0.378	1.00 (0.99-1.01)	0.658
CLIF-C AD score	1.03 (1.02-1.05)	**<0.001**		
MELD-score at admission	1.05 (1.04-1.07)	**<0.001**	1.05 (1.03-1.07)	**<0.001**
AD at admission				
No	Reference		Reference	
Yes	2.01 (1.42-2.84)	**<0.001**	1.91 (1.29-2.84)	**<0.001**
ACLF status				
No ACLF	Reference		Reference	
ACLF at time of AKI diagnosis	1.95 (1.46-2.59)	**<0.001**	0.79 (0.52-1.20)	0.270
ACLF development	3.13 (2.22-4.41)	**<0.001**	1.25 (0.82-1.91)	0.305
AKI etiology				
Pre-renal AKI	Reference		Reference	
HRS-AKI	1.55 (1.20-2.00)	**<0.001**	1.01 (0.74-1.38)	0.945
Other/mixed	1.05 (0.81-1.37)	0.740	1.05 (0.76-1.45)	0.761
AKI response end of admission				
Partial/Complete response	Reference		Reference	
No response/Progression	3.68 (2.95-4.59)	**<0.001**	3.41 (2.57-4.53)	**<0.001**
AKI stage				
1	Reference		Reference	
2	1.03 (0.95-1.79)	0.103	1.52 (1.06-2.17)	**0.023**
3	1.33 (1.06-1.68)	**0.014**	1.05 (0.72-1.38)	0.982

Univariate and multivariate cause specific regression analyses were performed to identify independent predictors of 1-year mortality. Results are presented as hazard ratios with 95% confidence intervals, p-values <0.05 were considered statistically significant. Abbreviations: CLIF-C AD score, Chronic Liver Failure Consortium Acute Decompensation score; AKI, acute kidney injury; AD, acute decompensation; MELD, Model For End-Stage Liver Disease; ACLF, acute-on-chronic liver failure; HRS-AKI, hepatorenal syndrome-acute kidney injury; CI, confidence interval; OR, odds ratio.
The bold value indicates the significant associations.

Table S7. Patient characteristics and mortality outcomes of patients diagnosed with AKI before and after 2015

	Hospitalization ≤2015 (n=263)	Hospitalization > 2015 (n=409)	p-value
Age	59 [52-65]	62 [54-68]	<0.001
MELD-score	24 [23-25]	24 [23-25]	0.384
AKI etiology			
Pre-renal AKI	78 (30)	137 (33)	0.310
HRS-AKI	65 (25)	158 (39)	**<0.001**
Other/mixed	120 (45)	114 (29)	**<0.001**
AKI stage			
1	139 (53)	228 (56)	0.476
2	28 (11)	60 (15)	0.160
3	96 (36)	121 (29)	0.064
28 days mortality	77 (29)	153 (37)	**0.031**
90 day mortality	124 (47)	195 (48)	0.937
1 year mortality	139 (53)	215 (53)	1.000

Data are presented as number (%) for categorical variables and median [IQR] for continuous variables. Comparisons between patients hospitalized ≤2015 and >2015 were performed using the Chi-square test, p-values <0.05 were considered statistically significant. Abbreviations: MELD, Model For End-Stage Liver Disease; AKI, acute kidney injury; HRS-AKI, hepatorenal syndrome-acute kidney injury.
